# Tolerability of a new amino acid-based formula for children with IgE-mediated cow’s milk allergy

**DOI:** 10.1186/s13052-021-01096-3

**Published:** 2021-07-03

**Authors:** Rita Nocerino, Carmen Di Scala, Serena Coppola, Veronica Giglio, Laura Carucci, Linda Cosenza, Luana Voto, Anna Maria Iannicelli, Anna Luzzetti, Roberto Berni Canani

**Affiliations:** 1 Department of Translational Medical Science, University of Naples Federico II, Naples, Italy; 2 ImmunoNutritionLab at CEINGE Advanced Biotechnologies, University of Naples Federico II, Naples, Italy; 3 European Laboratory for the Investigation of Food-Induced Diseases, University of Naples, Federico II, Naples, Italy; 4 Task Force on Microbiome Studies, University of Naples Federico II, Via S. Pansini 5, 80131 Naples, Italy

**Keywords:** Infant formula, Hypoallergenic formula, Food allergy, Diet therapy

## Abstract

**Background:**

Amino acid-based formula (AAF) is a relevant dietary strategy for paediatric patients affected by cow’s milk allergy (CMA). The present study was designed to evaluate the hypoallergenicity of a new AAF in children with immunoglobulin (Ig)E-mediated CMA.

**Methods:**

According to the criteria provided by the American Academy of Pediatrics Subcommittee on Nutrition and Allergic Diseases, we designed a prospective trial in CMA children (aged 1–36 months) aimed to demonstrate the hypoallergenicity of the new AAF in 90% of subjects with 95% confidence during the double-blind, placebo-controlled challenge (DBPCFC). A skin prick test (SPT) with the new AAF was also performed.

**Results:**

Twenty-nine children [all Caucasian, 55.2% male, mean age (±SD) 16.9 ± 5.7 months] were enrolled. The SPT and the DBPCFC with the new AAF were negative in all study subjects.

**Conclusions:**

The study results support the hypoallergenicity of the new AAF. This formula could be considered an additional dietary option for non-breastfed children affected by CMA.

**Trial registration:**

The trial was registered in the ClinicalTrials.gov Protocol Registration System (ID number: NCT03909113).

**Supplementary Information:**

The online version contains supplementary material available at 10.1186/s13052-021-01096-3.

## Introduction

With a 2.0 to 7.5% global prevalence, cow’s milk allergy (CMA) is the most widespread food allergy (FA) in the paediatric age [1–7]. The current standard of care for CMA is based on the strict dietary avoidance of cow’s milk protein-containing foods. For CMA infants, when breast milk is unavailable, the only remaining option is the use of a substitute formula, which is highly controlled for nutritional content and tolerance in these particular patients [8–15].

Amino acid-based formula (AAF) has been proposed for paediatric patients with severe CMA, multiple food allergies, eosinophilic esophagitis, food protein-induced enterocolitis syndrome, and severe eczema, or when the extensively hydrolyzed formula is not tolerated [16–20].

The American Academy of Pediatrics (AAP) Subcommittee on Nutrition and Allergic Diseases established criteria to determine the hypoallergenicity of any formula intended for children with CMA by demonstrating tolerance in 90% of children with CMA with a 95% confidence interval [22]. The present study was designed to evaluate the hypoallergenicity of a new AAF in children with confirmed immunoglobulin (Ig)E-mediated CMA.

## Methods

### Study design and study population

This prospective trial was conducted from March 2019 to March 2020 on patients aged 1–36 months with sure diagnosis of IgE-mediated CMA consecutively observed at tertiary centre for paediatric allergy.

The CMA diagnosis was confirmed in all subjects by the results of double-blind, placebo-controlled food challenge (DBPCFC) performed in the last 12 weeks*.* We excluded subjects aged < 1 month and > 36 months, breastfed infants, children with other food allergies, other allergic diseases, evidence of non-IgE-mediated CMA, eosinophilic disorders of the gastrointestinal tract, chronic systemic diseases, congenital cardiac defects, active tuberculosis, autoimmune diseases, immunodeficiency, chronic inflammatory bowel diseases, celiac disease, cystic fibrosis, metabolic diseases, malignancy, chronic pulmonary diseases, malformations of the gastrointestinal and/or respiratory tract, use of systemic antibiotics or anti-mycotic drugs during 4 weeks before study entry, presence of CMA-related symptoms in the previous 2 weeks, investigator’s uncertainty about the willingness or ability of the subject to comply with the protocol requirements, and participation in any other studies involving investigational or marketed products concomitantly or within 2 weeks prior to entry into the study.

### Ethics

The study protocol, the subject information sheet, the informed consent form, and the clinical chart were reviewed and approved by the Ethics Committee of the University of Naples Federico II. The study was conducted in accordance with the Helsinki Declaration (Fortaleza revision 2013), the Good Clinical Practice Standards (CPMP/ICH/135/95), and the current Decree-Law 196/2003 regarding personal data and all the requirements set out in the European regulations on this subject. The study was registered in the ClinicalTrials.gov Protocol Registration System with the ID number NCT03909113.

### Data collection

At baseline, after obtaining informed consent from the parents/tutors of each subject, the clinical status of the patients was carefully assessed by a multidisciplinary team composed of paediatricians, paediatric allergists, paediatric nurses, and dietitians to exclude those with concomitant comorbidities. Infectious diseases or other conditions were ruled out by means of a complete physical examination, including vital signs, neurological status, body growth pattern, nutritional status, hydration, skin evaluation, otoscopy, evaluation of oral cavity, respiratory/abdomen/lymph node examination. At enrolment, anamnestic, demographic, anthropometric, and clinical data (including data related to CMA), as well as information on sociodemographic factors, were obtained from the parents of each child, collected in a specific clinical chart, and entered into the study database.

Then, a skin prick test (SPT) with the new AAF was performed. Briefly, the skin prick test was performed with the new AAF reconstituted according to the manufacturer’s instructions. The new AAF was applied to the patient’s volar forearm. Skin prick tests were performed using a 1-mm single peak lancet (ALK, Copenhagen, Denmark) with histamine dihydrochloride (10 mg/ml) and an isotonic saline solution (NaCl 0.9%) as positive and negative controls, respectively. Reactions were recorded on the basis of the largest diameter (in millimetres) of the wheal-and-flare reaction at 15 min. The SPT result was considered “positive” if the wheal was 3 mm or larger, without reaction to the negative control.

Subsequently, the patients underwent the DBPCFC with the new AAF or the placebo formula (namely, the formula previously given to the child as part of the child’s successful elimination diet before study inclusion) introduced in a random order, as previously described [23].

We created a computer-generated randomization list of participant numbers indicating the order in which each study formula was used in the oral food challenge (OFC). Randomization and preparation of the challenges were performed by an independent dietician not directly involved in the study and in the patient’s care. In addition, bottles were covered by a paper sheet so that they were not distinguishable. The investigator, the nursing staff, and the family were therefore not informed of what formula the child was being fed.

Before each OFC day, the investigator ensured that the child did not present any clinical abnormalities and had stopped all medications, including anti-histamines, that could have interfered with the administration of the OFC. Subjects were eating nothing for 1 h with allowance for light meals 2 h prior to each session of OFC.

Briefly, every 20 min, successive doses (0.5, 1, 3, 10, 30, 50 and 100 mL) were administered in a blinded manner under medical supervision. The infants were observed for 2 h after the final dose and then discharged. In the case of a positive OFC, at any testing dose, the patient was treated as deemed necessary by the investigator and remained under observation until symptom resolution.

If patients did not show any symptoms within the first 24 h, to assess long-term tolerance and reveal any false-negative results to the challenges, parents administered one single top dose (about 200 ml) of the tested formula (new AAF or placebo) to the patients every day at home for 7 days (7-day home feeding period), and parents were instructed not to introduce any new foods. In addition, an emergency treatment plan and prescriptions for emergency medications were provided to the parents. If any symptoms occurred during this period, the subjects returned to the outpatient clinic on the same day. During the 7-d home feeding period, parents were invited to record daily the following: the total amount of formula ingested by the subject; the types of foods eaten; the presence and severity of vomiting, diarrhoea, rash, runny nose, wheezing, or any other symptoms (rated as mild, moderate, or excessive); the number of bowel movements and stool colour, consistency and odour; any adverse or serious adverse events; and the formula acceptability by their child, from very unsatisfied to very satisfied. After a 7-day home feeding period of the new AAF or placebo administration, the patients were examined, and the parents were interviewed at the centre. To rule out a false-negative challenge result, parents contacted the centre if any symptoms occurred in the following 7 days after the OFC procedures. The challenge was considered negative if the patient tolerated the entire challenge, including the observation period.

All objective and subjective symptoms were assessed simultaneously by experienced paediatric allergists and were registered using a standardized symptom score [23, 24] (Supplementary Table 1).

The new AAF was provided by the study sponsor and was reconstituted according to the manufacturer’s instructions. The composition of the new AAF is described in Table 1.

**Table 1:** Composition of the study formula

		100 g	100 mL at 13% w/v
**Calories**	**kJ**	**1974**	**256**
	**kcal**	**471**	**61**
**Total fat**	**g**	**21.0**	**2.7**
Saturated fat	g	7.8	1.0
Monounsaturated fat	g	9.8	1.3
Polyunsaturated fat	g	3.0	0.4
**Total carbohydrate**	**g**	**58.2**	**7.6**
Sugars	g	0.0	0.0
**Protein**	**g**	**12.2**	**1.6**
**Salt**	**g**	**0.41**	**0.05**
**Minerals**			
Sodium	mg	165	21
Potassium	mg	570	74.1
Chloride	mg	300	39
Calcium	mg	460	59.8
Phosphorus	mg	295	38.4
Magnesium	mg	42	5.46
Iron	mg	6.9	0.90
Zinc	mg	7.1	0.92
Copper	g	420	55
Iodine	g	99	12.9
Manganese	mg	0.41	0.05
Fluorine	mg	0.3	0.04
Molybdenum	g	14.5	1.9
Chromium	g	14.5	1.9
Selenium	g	9.0	1.2
**Vitamins**			
Vitamin A	μg RE	550	71.5
Vitamin D	μg	8.0	1.0
Thiamin	mg	0.5	0.065
Riboflavin	mg	0.8	0.10
Niacin	mg	5.4	0.70
Vitamin B6	mg	0.7	0.09
Pantothenic Acid	mg	3.0	0.39
Biotin	μg	20	2.6
Folic Acid	μg	75	9.75
Vitamin B12	μg	2.1	0.27
Vitamin C	mg	63	8.2
Vitamin K	μg	60	7.8
Vitamin E	mg	10	1.3
**Other nutrition facts**			
Choline	mg	98	13
Inositol	mg	20	2.6
L-carnitine	mg	17.9	2.3
Taurin	mg	40	5.2
Linoleic Acid (LA)	mg	2900	377
α-linolenic Acid (ALA)	mg	294	38
Maltodextrins	g	42.3	5.5
**Nucleotides**			
Adenosine-5′-monophosphate	mg	6.9	0.9
Cytidine-5′-monophosphate	mg	3.8	0.5
Guanosine-5′-monophosphate	mg	1.3	0.2
Inosine −5′-monophosphate	mg	2.5	0.3
Uridine - 5′-monophosphate	mg	4.5	0.6
**Osmolarity**	mOsmol/l	216	

The composition of the new amino acid based formula was fully in line with the composition of other commercially available amino acid based formulas and with the actual recommendation for energy requirement provided by European Food Safety Authority (reference #27)

**Fig. 1:**
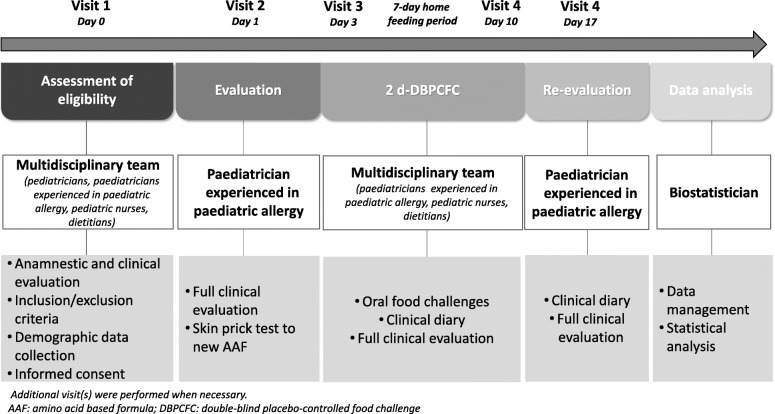
The design of the study

All study procedures and assessments were performed as shown in Fig. 1.

### Study outcome

The primary study outcome was the evaluation of the hypoallergenicity of the new AAF paediatric patients with IgE-mediated CMA.

#### Sample size

The sample size was calculated according to the AAP guidelines for clinical testing of hypoallergenic formulas [22]. In a study with a binomial outcome (reaction versus no reaction), the sample size can be determined by calculating a binomial confidence interval (CI) for p, the probability of having a reaction. The number of subjects needed to project with 95% confidence (one-sided interval) that less than 10% of infants will react to the product is 29 consecutive subjects if no clinical reactions are observed. These sample size estimates were derived based on binomial distribution techniques using Wald’s method for deriving confidence intervals for single proportions (software used: R Version 3.1.0–The R foundation for statistical computing).

#### Statistical analysis

The Kolmogorov-Smirnov test was used to determine whether variables were normally distributed. Descriptive statistics were reported as the means and standard deviations for continuous variables, and discrete variables were reported as the number and proportion of subjects with the characteristic of interest. All data were collected in a dedicated database and analysed by a statistician blinded to patient group assignment using SPSS for Windows (SPSS Inc., version 23.0, Chicago, IL).

## Results

**Fig. 2:**
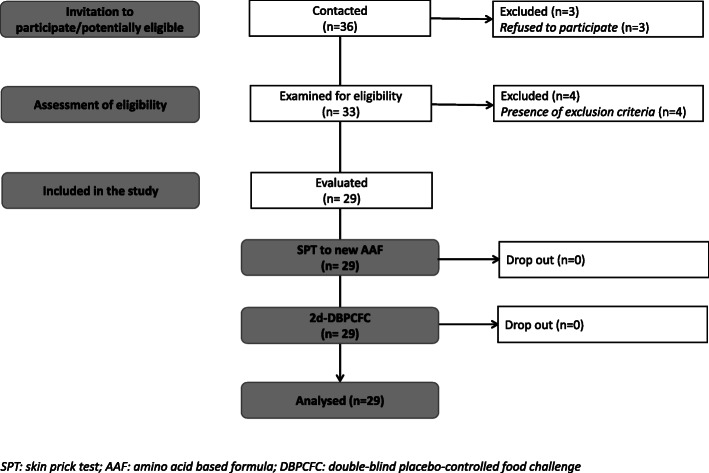
Flow of the children through the study

The flow of the subjects during the study is reported in Fig. 2.

A total of 36 consecutively potentially eligible paediatric patients were contacted and invited to participate in the study. Three subjects refused to participate; thus, 33 patients were examined for eligibility, and 4 were excluded because of the presence of exclusion criteria, leaving a total of 29 children included. All study subjects [all Caucasian, 55.2% male, mean age (±SD) at enrolment 16.9 ± 5.7 months] were from families of middle socioeconomic status and lived in urban areas. All subjects were weaned at enrolment and were receiving a cow’s milk protein-free diet according to age-related energy requirements. The formulas that the subjects were receiving at study entry were extensively hydrolyzed casein formula containing the probiotic *Lactobacillus rhamnosus* GG (37.9%), hydrolyzed rice formula (20.7%), extensively hydrolyzed whey formula (17.2%), soy formula (10.3%), or AAF (13.8%).

The main demographic and clinical features of the study subjects at enrolment are depicted in Table 2**.** The SPT with the new AAF was negative in all study subjects. Similarly, all children passed the DBPCFC with the new AAF.

**Table 2:** Baseline demographic and clinical characteristics of study population

PATIENT ID	SEX	TYPE OF DELIVERY	GESTATIONAL AGE	FAMILIAL RISK OF ALLERGY	AGE AT CMA DIAGNOSIS	AGE (months)	WEIGHT (kg)	LENGTH (cm)	HEAD CIRCUMFERENCE (cm)	TOTAL SERUM IgE (kU/l)	POSITIVE SKIN PRICK TEST TO COW MILK	ELICITING DOSE (POSITIVE OFC AT CMA DIAGNOSIS)	GI SYMPTOMS	CUTANEOUS SYMPTOMS	RESPIRATORY SYMPTOMS
						AT ENROLLMENT							AT CMA DIAGNOSIS		
001	F	SPONTANEOUS	At term	Yes	2	7	9.30	71.2	46	141	Yes	2nd	No	Yes	No
002	F	CAESAREAN	At term	No	5	9	9.55	75	46.3	125	Yes	2nd	Yes	Yes	No
003	F	SPONTANEOUS	At term	Yes	8	10	8.85	74	48	334	Yes	3rd	No	Yes	Yes
004	M	CAESAREAN	At term	Yes	2	11	9.60	76.2	46.3	734.5	Yes	1st	No	Yes	No
005	M	SPONTANEOUS	At term	Yes	5	12	9.85	76.5	47	162	Yes	2nd	No	Yes	No
006	F	SPONTANEOUS	At term	Yes	1	12	10.20	73	46.9	180	Yes	2nd	No	Yes	No
007	M	CAESAREAN	At term	Yes	5	12	10.55	74.2	46.7	153.6	Yes	2nd	No	Yes	No
008	F	SPONTANEOUS	At term	No	3	13	10.50	74	47.2	100.8	Yes	3rd	Yes	No	No
009	M	CAESAREAN	At term	Yes	3	13	9.40	74.1	47	229	Yes	1st	Yes	No	No
010	M	CAESAREAN	At term	Yes	1	14	11.20	77	47.3	311	Yes	1st	Yes	No	No
011	M	SPONTANEOUS	At term	No	6	14	11.85	76.2	47.6	292.1	Yes	2nd	No	Yes	No
012	M	CAESAREAN	At term	Yes	7	15	13.10	75.2	47.7	645.3	Yes	3rd	Yes	Yes	No
013	F	SPONTANEOUS	At term	No	1	15	12.60	78.3	47.2	75	Yes	2nd	Yes	Yes	No
014	F	CAESAREAN	At term	Yes	1	15	11.20	76	47.3	56	Yes	3rd	Yes	Yes	No
015	M	CAESAREAN	At term	No	1	16	11.00	77	47.2	49.7	Yes	3rd	Yes	Yes	No
016	F	SPONTANEOUS	At term	Yes	2	16	12.25	76	47.3	421	Yes	1st	No	No	Yes
017	F	SPONTANEOUS	At term	Yes	1	16	12.90	76.5	47.4	407.5	Yes	1st	No	No	Yes
018	M	SPONTANEOUS	At term	Yes	1	16	12.10	78.2	47.4	187	Yes	2nd	No	Yes	No
019	M	CAESAREAN	At term	No	1	17	12.70	78	47.3	343	Yes	1st	Yes	Yes	No
020	M	CAESAREAN	At term	No	1	19	12.30	83	47.5	176.5	Yes	1st	No	Yes	No
021	F	CAESAREAN	At term	Yes	3	22	13.10	85	48.3	429	Yes	2nd	Yes	Yes	No
022	M	SPONTANEOUS	At term	Yes	4	22	12.40	86.2	48.4	162	Yes	2nd	No	Yes	No
023	F	CAESAREAN	At term	Yes	6	23	13.25	83	48.6	98	Yes	3rd	No	Yes	No
024	M	SPONTANEOUS	At term	Yes	3	24	12.90	88.3	48.7	29.8	Yes	3rd	No	Yes	No
025	M	SPONTANEOUS	At term	Yes	2	24	13.00	87.9	48.9	461	Yes	2nd	Yes	No	No
026	M	CAESAREAN	At term	Yes	3	25	13.00	86.2	49.5	288.2	Yes	2nd	Yes	No	No
027	M	SPONTANEOUS	At term	Yes	7	25	13.60	84.6	49.6	209	Yes	3rd	No	Yes	No
028	F	SPONTANEOUS	At term	No	3	26	13.80	88.7	49.4	361	Yes	2nd	Yes	No	No
029	F	SPONTANEOUS	At term	No	2	28	13.75	85.1	48.6	413.3	Yes	1st	No	Yes	No

The new study formula was well accepted by the children, as confirmed by daily parental records and the very high adherence rate of subjects during the OFC and the 7-day open phase.

## Discussion

This is the first study investigating the tolerance to this new AAF in paediatric patients with challenge-proven IgE-mediated CMA. The study provided 95% confidence that more than 90% of subjects with CMA tolerate the new AAF, thus demonstrating the hypoallergenicity of this formula.

The hypoallergenicity of the new AAF was further confirmed by the SPT result, which was negative in all study subjects.

The amino acid-based formula is considered the only completely non-allergenic formula. It can be the best effective dietary option in patients who do not respond to extensively hydrolyzed formulas, in patients with anaphylaxis or with severe forms of CMA [13, 25–27].

This study presents several strengths. First, it was performed on a well-characterized population of children with previous challenge-proven IgE-mediated CMA followed by specialists at a tertiary paediatric allergy centre. Second, the methodology adopted in this study was rigorous. Nonetheless, this study has limitations. Our data cannot be generalized to children with conditions that were reasons for exclusion from the study. Another limitation of our study is the lack of results of the longer evaluation of body growth of the enrolled patients, but the composition of the new AAF was fully in line with the composition of other commercially available AAFs and with the actual recommendation for energy requirement provided by the European Food Safety Authority [28]. Thus, we can assume that this new AAF could provide normal body growth for paediatric patients affected by CMA. To better assess this aspect, future studies are advocated. Finally, as in other studies conducted on AAFs, further studies are required to investigate the long-term effects of this dietary treatment on the time of immune tolerance acquisition in children with CMA.

In conclusion, the new AAF meets the AAP criteria for hypoallergenicity, is well tolerated in short-term use and constitutes an additional safe option among the various formulas already available for the dietary management of non-breastfed children with CMA.

## Supplementary Information

**Additional file 1: Supplementary Table 1.** Pre-specified scale of allergic symptoms used to assess reactions during the double-blind placebo-controlled food challenge.

## Data Availability

The datasets used and/or analysed during the current study are available from the corresponding author on reasonable request.

## Abbreviations

CMA: Cow’s milk allergy
CMP: Cow milk protein
IgE: Immunoglobulin E
AAF: Amino acid-based formula
AAP: American Academy of Paediatrics
DBPCFC: Double-blind, placebo-controlled challenge
OFC: Oral food challenge
SPT: Skin prick test
SD: Standard deviation
CI: Confidence interval
