# Supplementary material for: Tolerability of a new amino acid-based formula for children with IgE-mediated cow’s milk allergy
Source: Ital J Pediatr. 2021 Jul 3;47:151. doi: 10.1186/s13052-021-01096-3 (PMC8254988; doi:10.1186/s13052-021-01096-3)
Supplement: Supplementary file 1 — Additional file 1: Supplementary Table 1. Pre-specified scale of allergic symptoms used to assess reactions during the double-blind placebo-controlled food challenge. [file 13052_2021_1096_MOESM1_ESM.docx]

**Supplementary Table 1.** Pre-specified scale of allergic symptoms used to assess 7 reactions during the double-blind placebo-controlled food challenge.

| **Mild symptoms** | **Moderate symptoms** | | **Severe symptoms** |
| --- | --- | --- | --- |
| **SKIN** | | | |
| *Pruritus* | | | |
| Occasional scratching | Scratching continuously for > 2 minutes at a time | | Hard, continuous scratching excoriations |
| *Urticaria/Angioedema* | | | |
| Less than 3 hives, or mild lip oedema | < 10 hives but >3, or significant lip or face oedema | | Generalized involvement |
| *Rash* | | | |
| Few areas of faint erythema | Areas of erythema, macular and raised rashes | | Generalized marked erythema (>50%), extensive raised lesions (> 25%), vesiculation and/or piloerection |
| **UPPER RESPIRATORY** | | | |
| *Sneezing/Itching* | | | |
| Rare bursts, occasional sniffing | Bursts < 10, intermittent rubbing of nose, and/or eyes and/or external ear canals (or frequent sniffing) | | Continuous rubbing of nose and/or eyes, periocular swelling and/or long bursts of sneezing (persistent rhinorrhoea) |
| *Nasal Congestion* | | | |
| Some hindrance to breathing | Nostrils feel blocked, frequent breathing through the mouth | | Nostrils occluded |
| *Rhinorrhoea* | | | |
| Occasional sniffling | Frequent sniffling, requires tissues | | Nose runs freely despite sniffling and tissues |
| **LOWER RESPIRATORY** | | | |
| *Laryngeal* | | | |
| (>3 discrete episodes of) throat clearing, (or) occasional cough, (or persistent throat tightness/pain) | Hoarseness, frequent dry cough | | (inspiratory) stridor |
| *Wheezing* | | | |
| Expiratory wheezing to auscultation | Inspiratory and expiratory wheezing | | Dyspnoea, use of accessory muscles, audible wheezing |
| **GASTROINTESTINAL** | | | |
| *Subjective Gastrointestinal Complaints* | | | |
| Complaints of nausea or abdominal pain, itchy mouth/(throat), no change in activity | Frequent complaints of nausea or pain with (normal) decreased activity | | Patient in bed, crying, notably distressed due to gastrointestinal symptoms (with decreased activity) |
| *Objective Gastrointestinal Complaints* | | | |
| 1 episode of emesis or diarrhoea | 2-3 episodes of emesis or diarrhoea or 1 of each | | >3 episodes of emesis or diarrhoea or 2 of each |
| **CARDIOVASCULAR/NEUROLOGICAL** | | | |
| Colour change, subjective response (weak, dizzy), mental status change or tachycardia | Decrease in blood pressure >20% from baseline (or significant change in mental status.) | Cardiovascular collapse, signs of impaired circulation (unconscious), bradycardia | |
